# Characterization and phylogenetic analyses of ten complete plastomes of *Spiraea* species

**DOI:** 10.1186/s12864-023-09242-3

**Published:** 2023-03-21

**Authors:** Shu-Dong Zhang, Kai Yan, Li-Zhen Ling

**Affiliations:** School of Biological Science and Technology, Liupanshui Normal University, Liupanshui, Guizhou, China

**Keywords:** *Spiraea*, Chloroplast genome, Phylogenetic analysis, Rosaceae

## Abstract

**Background:**

*Spiraea* is a genus of deciduous shrubs that contains 80-120 species, is mainly distributed in the Northern Hemisphere and has diversified in East Asia. *Spiraea* species are cultivated as ornamental plants and some are used in traditional herbal medicine. Based on morphological characteristics and genetic markers, phylogenetic classification exhibits low discriminatory power.

**Results:**

In present study, we assembled and characterized the chloroplast (cp) genomes of ten *Spiraea* species and comparatively analysed with five reported cp genomes of this genus. The cp genomes of the fifteen *Spiraea* species, ranging from 155,904 to 158,637 bp in length, were very conserved and no structural rearrangements occurred. A total of 85 protein-coding genes (PCGs), 37 tRNAs and 8 rRNAs were annotated. We also examined 1,010 simple sequence repeat (SSR) loci, most of which had A/T base preference. Comparative analysis of cp genome demonstrated that single copy and non-coding regions were more divergent than the inverted repeats (IRs) and coding regions and six mutational hotspots were detected. Selection pressure analysis showed that all PCGs were under purifying selection. Phylogenetic analysis based on the complete cp genome data showed that *Spiraea* formed a monophyletic group and was further divided into two major clades. Infrageneric classification in each clade was supported with a high resolution value. Moreover, the phylogenetic trees based on each individual mutational hotspot segment and their combined dataset also consisted of two major clades, but most of the phylogenetic relationships of interspecies were not well supported.

**Conclusions:**

Although the cp genomes of *Spiraea* species exhibited high conservation in genome structure, gene content and order, a large number of polymorphism sites and several mutation hotspots were identified in whole cp genomes, which might be sufficiently used as molecular markers to distinguish *Spiraea* species. Phylogenetic analysis based on the complete cp genome indicated that infrageneric classification in two major clades was supported with high resolution values. Therefore, the cp genome data of the genus *Spiraea* will be effective in resolving the phylogeny in this genus.

**Supplementary Information:**

The online version contains supplementary material available at 10.1186/s12864-023-09242-3.

## Background

*Spiraea* L. is a genus of deciduous shrubs of Rosaceae tribe Spiraeeae (subfamily Amygdaloideae), which is widespread in the Northern Hemisphere [1, 2]. East Asia has become the center of the diversity of this genus. For example, approximately 70 *Spiraea* species have been identified in China [3]. *Spiraea* has become a commercially important genus with ornamental and therapeutic properties. Many *Spiraea* species are applied in landscaping owing to the variations in morphological characteristics. For example, they have a high accumulated flower abundance and different flower colours in simple and compound inflorescences [2]. Moreover, representatives of the genus are widely used in conventional medicine, with several effective therapeutics for inflammation and malaria [2, 4]. To date, many chemical components (phenolic compounds, terpenoids, alkaloids, and steroids) have been isolated and characterized in species of *Spiraea* worldwide [2]. Among them, many substances have exhibited biological activities; thus *Spiraea* species have high potential as a valuable resource. For example, the medicinal use of *Spiraea* is confirmed in folk medicine [5, 6].

The genus *Spiraea* includes 80 to 120 species, and a portion of them are interspecific hybrid species due to open pollination during cultivation [7]. Infrageneric classifications in the genus *Spiraea* have been mainly determined on the basis of inflorescence morphology, which is divided into three sections (i.e., *Spiraea*, *Calospira* and *Chamaedryon*). Alternatively, section *Chamaedryon s.l*. was further separated into section *Sciadantha*, thus four sections were identified in some treatments [8]. For decades, a few plastid or nuclear gene fragments and markers, including *trnL-trnF*, *matK*, *rpl20-rpl12*, *psbA-trnH*, *rps15-ycf1* and *trnS-trnG*, nuclear ribosomal DNA internal transcribed spacers (ITS) and AFLP marker, have been applied to assess phylogenetic relationships within the genus [8–11]. All these molecular phylogenetic analyses have indicated that *Spiraea* has been resolved as a monophyletic group. Although many taxa of the genus *Spiraea* were well separated from each other, some clades were not well supported based on the limited variable sites and were not consistent with the traditional taxonomic positions [8–11]. Thus, more genetic data are needed to gain insight into the phylogenetic relationships within *Spiraea*.

The chloroplast (cp) is a semiautonomous organelle with mainly maternally inherited DNA, which plays important roles in photosynthesis and carbon fixation in green plants [12, 13]. The cp genome (120 to 180 kb in size) has a characteristic circular structure and inverted repeat (IR) regions divide the cp genome into four parts: two IR regions, a large single copy (LSC) region and a small single copy (SSC) region [14]. These cp genomes can provide valuable information on genetic variations [15, 16] and have been widely used for species identification and phylogenetic analysis [17–19]. With the rapid development of next-generation sequencing (NGS) technology and reduction of costs, an increasing number of cp genome sequences have been obtained, which extends gene-based phylogenetics to phylogenomics. For example, we have previously used the whole plastid phylogenomic approach to reconstruct deep relationships of Rosaceae based on the representatives of 87 genera of this family [20]. Recently, Zhang et al. [21] reported 31 complete plastomes of *Rosa* species and comparatively analyzed the gene divergence of the plastomes in this genus.

To date, the complete cp genome sequences of five *Spiraea* species have been released in the GenBank database. In this study, the cp genomes of ten *Spiraea* species were newly sequenced using NGS technology and comparatively analysed with the five previously released cp genomes. We mainly analysed the structural features of cp genome, sequence variations, selective pressure and phylogenetic reconstructions. This study aimed to gain a comprehensive understanding of the cp genomes within *Spiraea*, to obtain the unambiguous phylogenetic relationships of the tested species within this genus.

## Results

### Chloroplast genome features of *Spiraea* species

**Fig. 1:**
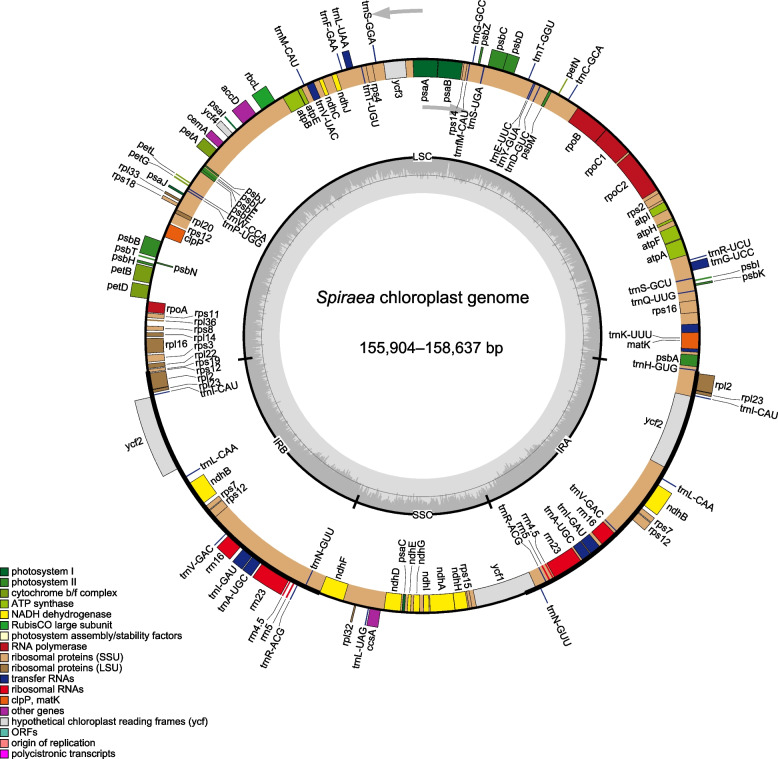
Chloroplast genome map of *Spiraea*. The grey inside circle indicates the GC level of every genomic position. Genes inside in the outer circle of genomic map are transcribed clockwise and vice versa. The different functional gene categories are shown in the different colors

In this study, the cp genomes of 10 *Spiraea* species were sequenced, assembled and submitted to GenBank (Table 1 and Additional file 1: Table S1). The ten new *Spiraea* cp genomes ranged from 155,904 (*S. salicifolia*) to 156,167 bp (*S. japonica*) in length and were very similar to most published cp genomes (Table 1). Among them, *S. insularis* had the largest genome size, which was 2,700 bp larger than that of *S. salicifolia* (Table 1). Each cp genome of ten *Spiraea* species was assembled into a single, circular DNA sequence and collinear to five previously published cp genomes in this genus (Fig. 1). The 15 cp genomes were all classical tetrad structures, containing a pair of IR copies with lengths of 26,335 (*S. elegans*) to 26,398 bp (*S. purpurea*), the LSC with a length of 84,319 (*S. salicifolia*) to 84,568 bp (*S. japonica*), and the SSC with a length of 18,866 (*S. hirsuta*) to 18,932 bp (*S. elegans*) (Fig. 1 and Table 1). This analysis also revealed that all fifteen *Spiraea* species had a very similar GC content, ranging from 36.69% (*S. hirsuta*) to 36.87% (*S. insularis*) (Table 1).

**Table 1:** Information of the complete cp genomes of 15 *Spiraea* species

**Species**	**Accession number**	**PCG**	**tRNA**	**rRNA**	**Total**	**Length of plastome (bp)**	**Length of LSC (bp)**	**Length of IRa (bp)**	**Length of SSC (bp)**	**GC content (%)**
Spiraea aquilegiifolia^*^	ON160000	85	37	8	130	155,927	84,355	26,339	18,894	36.75
*S. blumei*	MN418904	85	37	8	130	155,957	84,384	26,343	18,887	36.76
S. chinensis^*^	ON160001	85	37	8	130	155,913	84,347	26,336	18,894	36.77
S. elegans^*^	ON160002	85	37	8	130	155,975	84,375	26,334	18,932	36.74
S. henryi^*^	ON160004	85	37	8	130	155,931	84,362	26,335	18,899	36.75
S. hirsuta^*^	ON160003	85	37	8	130	155,953	84,401	26,343	18,866	36.75
*S. insularis*	MT412405	85	37	8	130	158,637	86,997	26,365	18,910	36.87
S. japonica^*^	ON194001	85	37	8	130	156,167	84,568	26,342	18,915	36.69
*S. mongolica* var.* mongolica*	MT732945	85	37	8	130	155,949	84,375	26,340	18,894	36.74
*S. mongolica* var. tomentulosa^*^	ON160006	85	37	8	130	155,915	84,340	26,335	18,905	36.75
S. purpurea^*^	ON160007	85	37	8	130	156,156	84,469	26,398	18,891	36.81
S. salicifolia^*^	ON159999	85	37	8	130	155,904	84,319	26,349	18,887	36.75
S. tianschanica^*^	ON160005	85	37	8	130	155,965	84,411	26,341	18,872	36.73
*S. trilobata*	MW822176	85	37	8	130	155,981	84,417	26,343	18,878	36.75
*S.* ×* vanhouttei*	MZ981785	85	37	8	130	155,957	84,384	26,343	18,887	36.76

PCG indicates protein-coding gene. The species with the asterisk (*) indicate the newly sequenced ones

All 15 *Spiraea* cp genomes possessed 130 genes arranged in the same order, including 85 protein-coding genes (PCGs), 37 tRNAs and eight rRNAs (Fig. 1 and Table 1), which were classified into four categories based on their functions (Table 2). Of them, six PCGs, seven tRNA genes and all four rRNA genes were duplicated in IR regions, and the detailed information was shown in Table 2. In addition, the data revealed that eighteen genes contained introns, of which sixteen genes had only one intron and two genes (*ycf3* and *rps12*) contained two introns (Table 2). The *ycf3* gene entirely located in the LSC region, whereas the *rps12* gene was a trans-regional gene with the 5’-end in LSC and the 3’-end in the IR region (Fig. 1),which is similar to many other plants [21, 22].

**Table 2:** Gene information of 15 *Spiraea* chloroplast genomes

**Function of genes**	**Group of genes**	**Gene name**
Photosynthesis	Photosystem I	*psaA*, *psaB*, *psaC*, *psaI*,* psaJ*
	Photosystem II	*psbA*, *psbB*, *psbC*, *psbD*, *psbE*, *psbF*, *psbH*, *psbI*, *psbJ*, *psbK*, *psbL*, *psbM*, *psbN*, *psbT*,* psbZ*
	Cytochrome b/f complex	*petA, petB*^a^*, petD*^a^*, petG, petL, petN*
	ATP synthase	*atpA, atpB, atpE, atpF*^a^*, atpH, atpI*
	NADH-dehydrogenase	*ndhA*^a^, ndhB^*a^*, ndhC, ndhD, ndhE, ndhF, ndhG, ndhH, ndhI, ndhJ, ndhK*
	Large subunit Rubisco	*rbcL*
Protein synthesis and DNA-replication	Subunits of RNA polymerase	*rpoA, rpoB, rpoC1a, rpoC2*
	Ribosomal protein small subunit	*rps2*, *rps3*, *rps4*, *rps7*^a^, *rps8*, *rps11*, *rps12*^ac^, *rps14*, *rps15*, *rps16*^b^, *rps18*,* rps19*
	Ribosomal protein large subunit	*rpl2*^ab^, *rpl14*, *rpl16*^b^, *rpl20*, *rpl22*, *rpl23*^a^, *rpl32*, *rpl33*,* rpl36*
	Transfer RNAs	*trnA-UGC*^ab^, *trnC-GCA*, *trnD-GUC*, *trnE-UUC*, *trnF-GAA*, *trnfM-CAU*, *trnG-GCC*, *trnG-UCC*^b^, *trnH-GUG*, *trnI-CAU*^a^, *trnI-GAU*^ab^, *trnK-UUU*^b^, *trnL-CAA*^a^, *trnL-UAA*^b^, *trnL-UAG*, *trnM-CAU*, *trnN-GUU*^a^, *trnP-UGG*, *trnQ-UUG*, *trnR-ACG*^a^, *trnR-UCU*, *trnS-GCU*, *trnS-GGA*, *trnS-UGA*, *trnT-GGU*, *trnT-UGU*, *trnV-GAC*^a^, *trnV-UAC*^b^, *trnW-CCA*,* trnY-GUA*
	Ribosomal RNAs	*rrn4.5*^a^, *rrn5*^a^, *rrn16*^a^, *rrn23*^a^
Other genes	Maturase	*matK*
	Translation initiation factor	*infA*
	C-type cytochrome synthesis gene	*ccsA*
	Acetyl-CoA-carboxylase	*accD*
	Inner membrane protein	*cemA*
	ATP-dependent protease	*clpP*^a^
Genes of unknown function	Conserved hypothetical gene	*ycf1*, *ycf2*^*a*^, *ycf3*^*c*^,* ycf4*

^a^indicates duplicated genes

^b^gene contains a single intron

^c^gene contains two introns

### Repeat sequence *analysis*

**Fig. 2:**
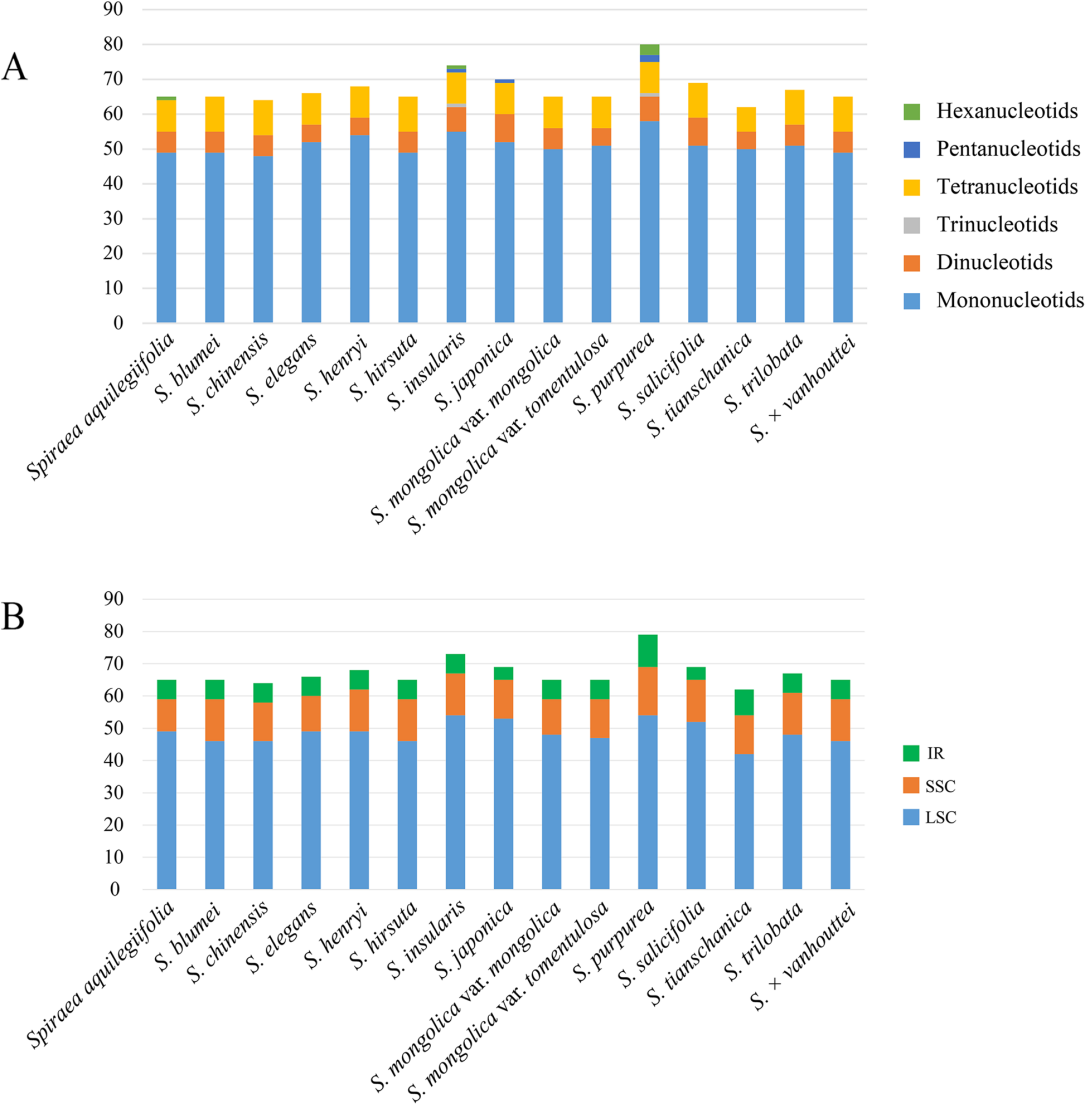
SSR loci analysis of fifteen *Spiraea*. (**A**) Number of six SSRs types; (**B**) Number of SSRs in LSC, SSC, and IR regions, respectively

Simple sequence repeats (SSRs) were analysed and a total of 1,010 SSRs in the 15 cp genomes were detected, ranging from 62 (*S. tianschania*) to 80 (*S. purpurea*) (Fig. 2A). The SSRs had a similar distribution pattern among the 15 *Spiraea* species, as shown in Fig. 2. Mononucleotides were the most frequent of the SSRs (72.5%-80.6%) followed by dinucleotides (6.2%-10.8%) and tetranucleotides (11.3%-15.6%). Trinucleotide, pentanucleotide and hexanucleotide sequences were very few across these cp genomes, and unevenly appeared in a few *Spiraea* species (Fig. 2A). These SSRs had a certain base preference and A/T repeats were the most common mononucleotide. In addition, dinucleotide repeats included a number of AT/AT repeat sequences and all tetranucleotide repeats were AAAT/ATTT (Additional file 2: Figure S1). SSRs were also found in all four regions of each cp genome. However, the results demonstrated that SSRs mainly distributed in the LSC region (67.5%-75.7%), followed by the SSC (15.3%-20.0%) region. In IR regions, only a few SSRs were detected (Fig. 2B).

Similar to the five reported cp genomes, the repeat types were consistent with those in the ten *Spiraea* species (Additional file 3: Figure S2). However, the number of each repeat type was different in these species. Palindromic repeats were the most abundant with a mean value of 24.7. Forward repeats were the second most abundant and most *Spiraea* species had 14-38 repeats, but *S. insularis* had the maximum number of forward repeats (more than 30) (Additional file 3: Figure S2). Reverse and complement repeats had the lower distributions in all 15 cp genomes. For example, only one complement repeat was found in each *Spiraea* cp genome (Additional file 3: Figure S2).

### Sequence divergent and selection pressure analysis

**Fig. 3:**
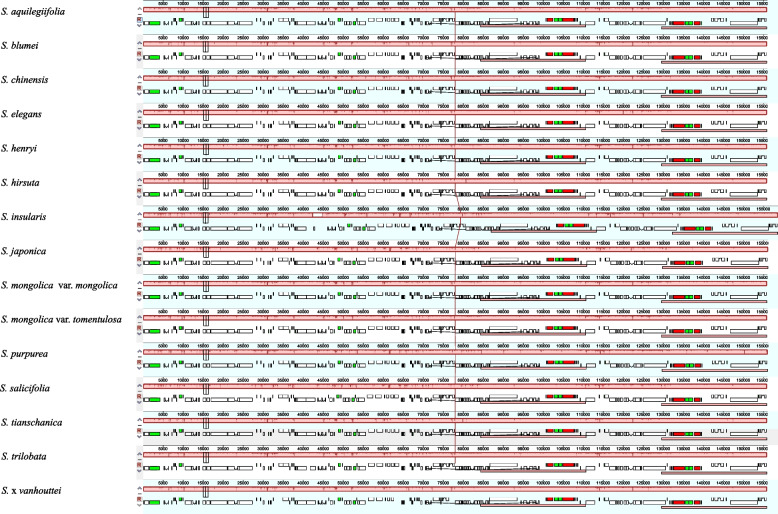
Colinear analysis of fifteen *Spiraea* species chloroplast genomes. The collinear blocks are marked with the same color

Mauve alignment analysis revealed that the whole cp genome sequences were highly homologous (Fig. 3). No structural rearrangement occurred in coding and noncoding regions of these cp genomes, suggesting that these 15 *Spiraea* species shared the same order and orientation of syntenic blocks (Fig. 3). Overall, the 15 *Spiraea* cp genomes were relatively conserved. Meanwhile, the cp genomes of the 15 *Spiraea* species were compared to analyse the overall sequence identity by the mVISTA program using the *S. aquilegiifolia* plastome as a reference (Additional file 4: Figure S3). The comparative results showed that the two IR regions were less divergent than the SC regions (Additional file 4: Figure S3). In addition, the noncoding regions showed more variations than the protein-coding regions (Additional file 4: Figure S3).

**Fig. 4:**
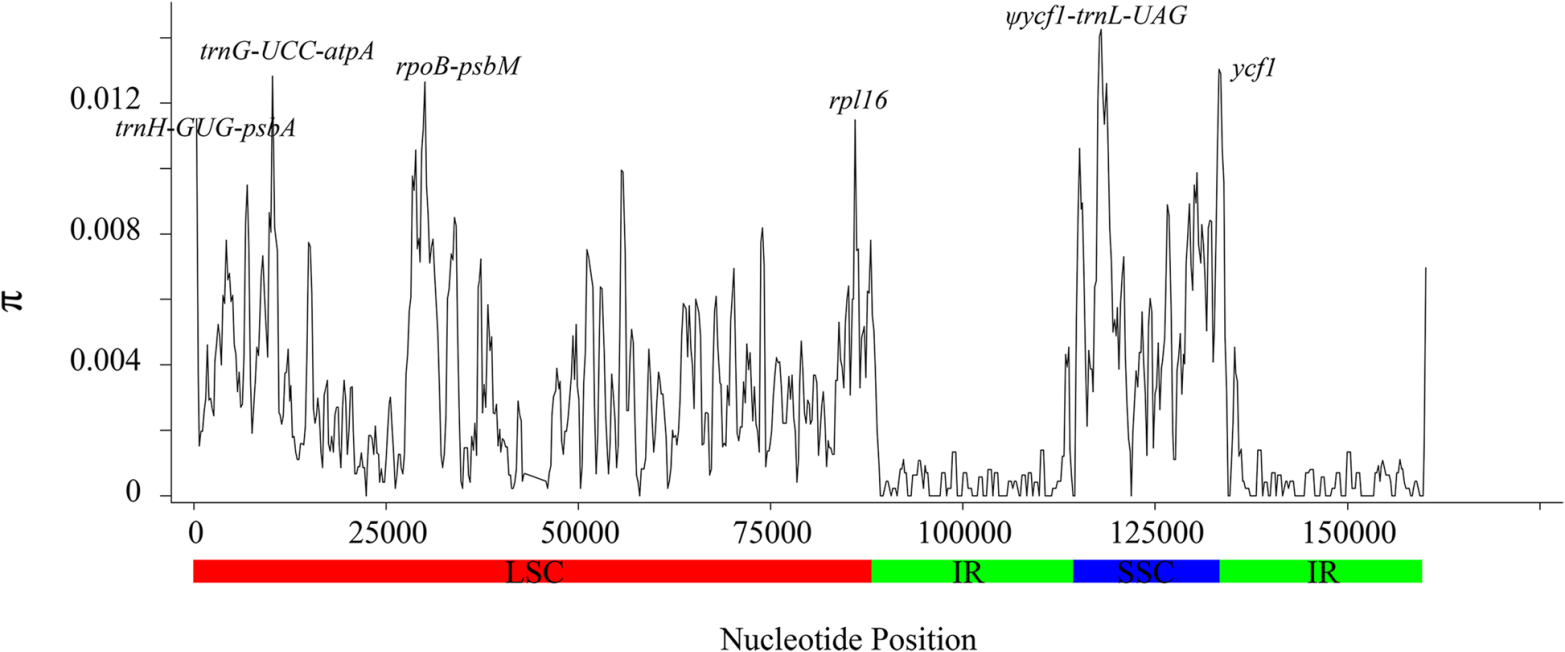
The $\pi$ values of the fifteen *Spiraea* chloroplast genome sequences by sliding window analysis (window length: 600 bp with step size of 200 bp)

To further understand the level of sequence divergence in the different regions of cp genomes, we calculated the nucleotide diversity ($\pi$) values within a 600-bp window (200 bp step size) across 15 *Spiraea* species. The $\pi$ values varied from 0 to 0.018, and the mean value was 0.0034 (Fig. 4), indicating that these sequences had high similarity. Then, six mutation hotspots, namely, *trnH-GUG-psbA*, *trnG-UCC-atpA*, *rpoB-psbM*, *rpl16*, *ψycf1-trnL-UAG* and *ycf1* were examined with a cutoff of 0.01. All hotspot regions were located in SC regions and only *ycf1* was in the coding region. Among them, the *ψycf1-trnL-UAG* region showed the highest divergence (Fig. 4).

To assess the selective pressure on the 79 distinct PCGs in the cp genomes of 15 *Spiraea* species, we calculated the rates of synonymous (Ks) and nonsynonymous (Ka) substitutions and the Ka/Ks ratio (Additional file 5: Table S2). The Ks values ranged from 0 to 0.0640, with a total mean value of 0.027 across all whole cp genomes. All the Ka/Ks ratios of 79 unique PCGs were below 1, indicating that strong purifying selection acted on these genes and that few amino acid changes occurred during evolution (Additional file 5: Table S2).

### Phylogenetic analysis of* Spiraea* species

**Fig. 5:**
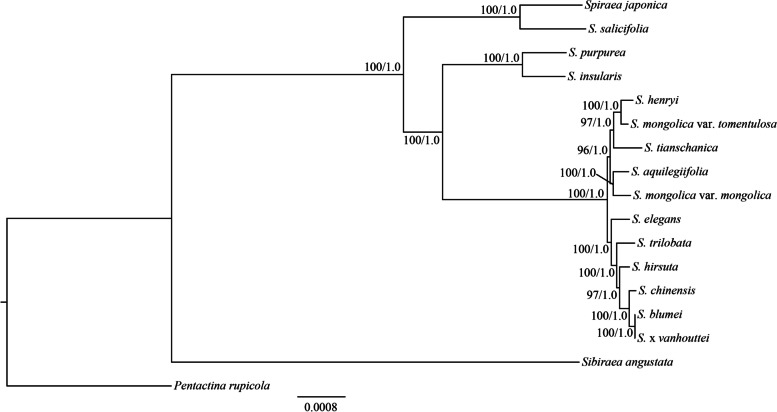
Phylogenetic tree of the fifteen *Spiraea* species inferred by maximum likelihood (ML) and Bayesian inference (BI) methods based on the complete plastomes. Numbers at nodes correspond to ML bootstrap percentages and BI posterior probabilities

In this study, cp genome sequence alignment of a total of 15 *Spiraea* species was used to reconstruct the Bayesian inference (BI) and maximum likelihood (ML) trees (Fig. 5). The topologies of the BI and ML trees were consistent and constituted a monophyletic group in *Spiraea* with very strong support (posterior probability (PP) = 1.00 for the BI tree and bootstrap value (BS) = 100% for the ML tree) (Fig. 5). In the two phylogenetic trees, two clades were constructed with a 100% BS and 1.0 PP supports. *Spiraea japonica* and *S. salicifolia* constituted one clade (Clade I) (BS = 100%, PP = 1.0), and the remaining 13 *Spiraea* species formed a large clade (Clade II). Of them, *S. purpurea* and *S. insularis* were clustered into one clade (Clade II-1) (BS = 100%, PP = 1.0), and the rest of the *Spiraea* species were clustered into another clade (Clade II-2) (Fig. 5). Additionally, infrageneric classification in each clade was supported with a high resolution value.

**Fig. 6:**
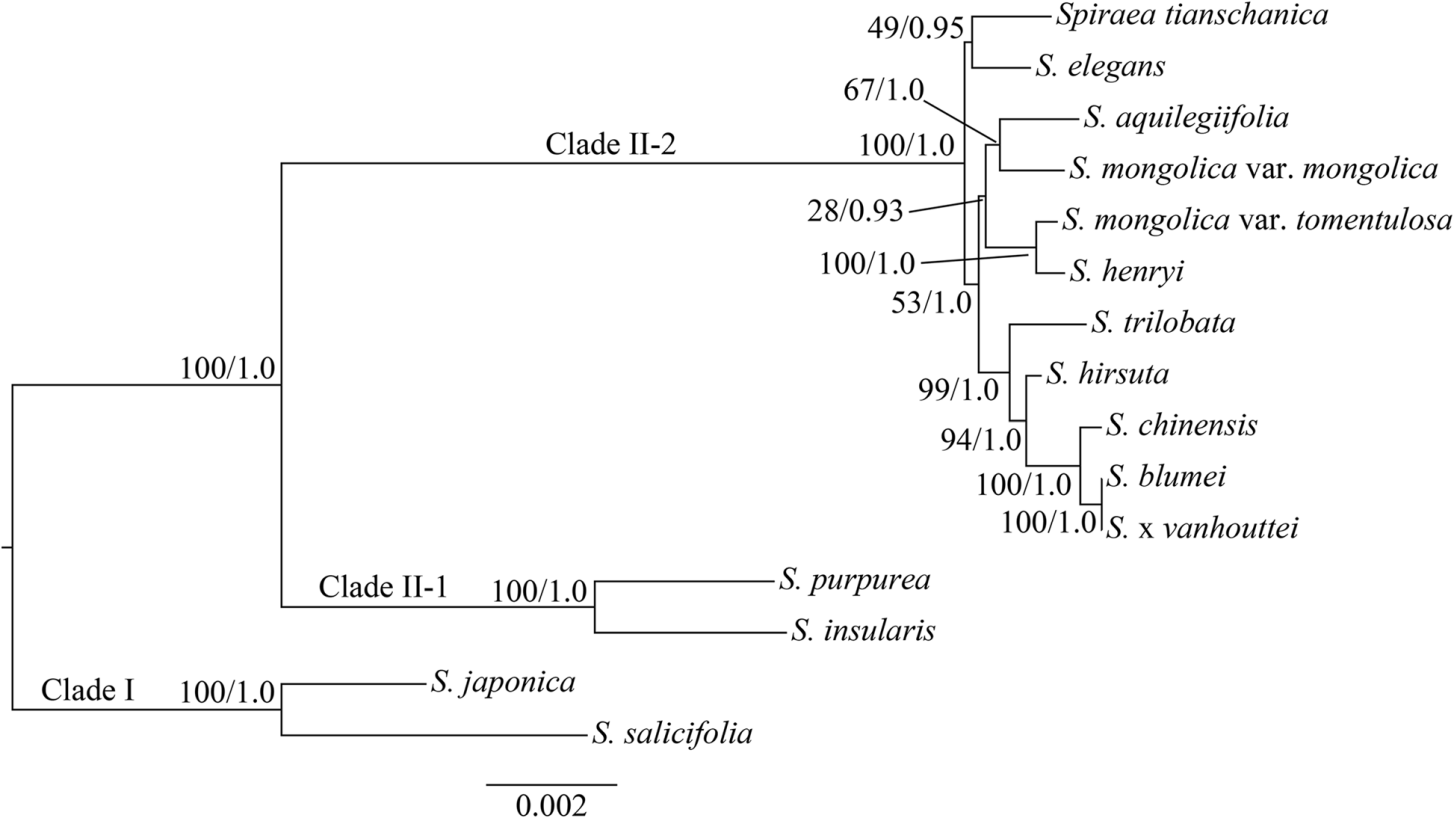
Phylogenetic tree of the fifteen *Spiraea* species inferred by maximum likelihood (ML) and Bayesian inference (BI) methods based on the combined six mutational hotspot regions. Numbers at nodes correspond to ML bootstrap percentages and BI posterior probabilities

In addition, we reconstructed the phylogenetic trees using each of six mutational hotspot regions based on the two methods (ML and BI). The results indicated that the topologies of the phylogenetic trees were identical for each variable region and also divided into two clades (Clade I and Clade II) (Additional file 6: Figure S4A-F). There was one exception in which *S. purpurea* and *S. insularis* were not clustered into one clade in the phylogenetic tree based on *trnH-GUG-psbA* (Additional file 6: Figure S4F). Among the 5 other hotspot regions, we found that the interspecies classification in Clade II-2 was not absolutely determined (Additional file 6: Figure S4A-E). When six hotspot regions were combined into one dataset, the generated phylogenetic tree indicated that the major relationships within the genus were well supported, but the relationship of *S. tianschanica* and *S. elegans* with low bootstrap support was different from that based on the whole cp genome dataset (Figs. 5 and 6).

## Discussion

In this study, we characterized the complete cp genomes of 10 *Spiraea* species and compared them with those of five available species within this genus. The results demonstrated that 15 *Spiraea* species had a conserved genome structure with characteristics of angiosperms. The cp genomes had the classical quadripartite structure of angiosperms (Fig. 1), which comprised two copies of IR region, one SSC region and one LSC region [23]. Then, the AT content was larger than that of GC in the cp genomes of 15 *Spiraea* species (Table 1), which is very universal in angiosperms [24, 25]. Of the 15 cp genomes of *Spiraea* species, the reported genome size of *S. insularis* was 2,700 bp longer than that of *S. salicifolia* (Table 1), which did not exceed the size range of typical angiosperm plastomes [26]. This size difference was mainly attributed to the length variation of the LSC region (Table 1), as reported previously [24]. Previous studies reported that repeats occur in genomes, in which many inversions might have occurred [27, 28]. Although some dispersed repeats were harboured in the cp genomes of the 15 *Spiraea* species, the gene order and orientation were unchanged. These results indicated that no inversions or rearrangement occurred in the tested plastomes (Figs. 1 and 3).

Using cp genomes to reconstruct plant phylogenies mainly involve structural rearrangement and sequence variation [27]. For example, many cp genomes of Campanulaceae [29] and Geraniaceae [30] plants are highly rearranged, which is significantly implied in altering the classification of these families. Previous studies have indicated that changes of gene content and inversions are regarded as the major mutation types of structural rearrangement [27, 31]. These mutation types are often made excellent characters for phylogenetic analysis. For example, the cpDNA inversion was examined in all tribes of Asteraceae, but this inversion was lacked in the subtribe Barnadesiinae of the tribe Mustisieae and all related families, which makes the Barnadesiinae as the basal plants in the Asteraceae [32]. In this study, the cp genomes of the tested 15 *Spiraea* species had the same gene content, the identical gene order and orientation, and lack of inversion. Obviously, our results indicated that lack of genome rearrangement was not used for the phylogenetic analysis of *Spiraea*.

In contrast, a number of SSRs were found in the cp genomes of 15 *Spiraea* species and most of sequence variations appeared in noncoding regions (Additional file 2: Figure S1 and Additional file 4: Figure S3). In addition, six mutational hotspots mainly located in intergenic regions. These highly divergent regions can be employed as potential DNA markers for studies on species identification and phylogenetic relationships [33–35]. The phylogenetic trees generated by individual mutational hotspot segments except *trnH-GUG-psbA* were divided *Spiraea* into two major clades with high support, which was consistent with cp genome result (Fig. 5 and Additional file 6: Figure S4A-E). However, previous studies based on the different cpDNA segments (*trnL-trnF*, *matK*, *rpl20-rpl12*, *psbA-trnH*, *rps15-ycf1* and *trnS-trnG*) have shown different topologies, in which the identical major clades were not generated, and the infrageneric phylogeny of *Spiraea* was also conflicted [8–11]. These phenomena might be attributed to the lower nucleotide diversity of these segments. Based on our analysis, the previous six cpDNA segments were not mutational hotspots and had lower $\pi$ values (Fig. 4). Similarly, *trnH-GUG-psbA* had the lowest $\pi$ value among six mutational hotspots (Fig. 4), and the topology based on this segment was different from those of 5 other segments (Additional file 6: Figure S4A-F). When we combined six highly variable regions as one dataset, the major interspecies relationships of *Spiraea* were well supported, except for *S. tianschanica* and *S. elegans* (Fig. 6). Therefore, the combined dataset did not have high discriminatory power compared to the cp genome data. In contrast, the phylogenetic tree generated by the cp genome not only robustly supported two major clades of 15 *Spiraea* species, but also showed high bootstrap or posterior probability values among interspecies *Spiraea* (Fig. 5). Altogether, these results demonstrated that cp genome data can effectively resolve the phylogenetic relationships within *Spiraea*.

## Conclusions

In this study, the cp genomes of 10 *Spiraea* species were newly sequenced and compared with those of 5 available *Spiraea* species obtained from the NCBI GenBank database. Our results indicated that the cp genomes of fifteen *Spiraea* species had a rather conserved genomic structure. Comparative analysis among these cp genome sequences identified 1,010 SSRs and they mainly located in the LSC region. Of them, mononucleotide repeats were the most abundant with a high A/T base preference. Although all the PCGs in the *Spiraea* cp genomes were under purifying selection, six gene-containing regions formed mutation hotspots. The phylogenetic reconstruction based on the complete cp genome data showed that *Spiraea* is a monophyletic group and is further divided into two major clades with high support. These results indicated that cp genome data will be great value for further resolution of the phylogeny of the genus *Spiraea*.

## Materials and methods

### Plant material and cpDNA sequencing

Ten *Spiraea* species in China were collected in the field: *S. aquilegiifolia* and *S. salicifolia* in the Nei Mongol Autonomous Region, *S. chinensis* and *S. henryi* in Hubei Province, *S. elegans* in Hebei Province, *S. hirsuta* in Shandong Province, *S. purpurea* and *S. japonica* in Sichuan Province, *S. tianschanica* in the Xinjiang Uygur Autonomous Region, *S. mongolica* var. *tomentulosa* in the Ningxia Hui Autonomous Region. These wild plants are not recorded as the national key protected plants and can be collected without permission. The voucher specimens of the ten species were identified by Shu-Dong Zhang and deposited in the herbarium of Kunming Institute of Botany, CAS (KUN) and the detailed information was shown in Additional file 1: Table S1. Genomic DNA of each species was extracted using the modified CTAB method from leaves dried by silica gel [20]. DNA concentration and quality were checked by a NanoPhotometer P330 (Implen GmbH, Munich, Germany) and 1% agarose gels. The DNA samples were fragmented to construct 350 bp library and sequenced according to the Illumina HiSeq 2500 protocol.

### Chloroplast genome assembly and annotation

The raw genome data was filtered and then de novo assembled using the GetOrganelle pipeline (https://github.com/Kinggerm/GetOrganelle). Dual Organellar GenoMe Annotator (DOGMA, http://dogma.ccbb.utexas.edu/) with manual adjustments was used to perform the gene annotation of the cp genomes, including PCGs, tRNAs and rRNAs. All tRNAs were further determined by the online tRNAscan-SE Search Service (http://lowelab.ucsc.edu/tRNAscan-SE/). The genome map was drawn using Organellar Genome DRAW (OGDRAW, http://ogdraw.mpimp-golm.mpg.de/). Geneious was used to analyse the GC level of each assembled cp genome.

### Repeat element analysis

Six types of simple sequence repeat (SSR) motifs, including mono-, di-, tri-, tetra-, penta-, and hexanucleotides, were detected using MISA-web (https://webblast.ipk-gatersleben.de/misa/), and the minimum thresholds were set to 10, 5, 4, 3, 3, and 3, respectively. Four kinds of repeats, including forwards, reverse, palindromic, and complementary repeats were identified using REPuter software [36].

### Comparative analysis of *Spiraea* plastomes

The plastomes of 15 *Spiraea* species were aligned with the MAFFT v.6.833 program [37] using the default settings. Subsequently, the sequence alignment was visualized using mVISTA [38] with the cp genome of *S. aquilegiifolia* as the reference. Cp genome homology and collinearity were analysed using Mauve software [39]. Moreover, a sliding window analysis was performed to compute the nucleotide diversity ($\pi$) of the cp genome sequences using DnaSP v. 5.0 [40]. The window length was set to 600 bp with a step size of 200 bp.

The coding sequence (CDS) of the PCGs from 15 *Spiraea* species was extracted and aligned by MAFFT v.6.833 [37]. And these alignments were used to calculate the ratio of nonsynonymous substitution (Ka) and synonymous substitution (Ks) by DnaSP v. 5.0 [40]. The different values of Ka/Ks indicate the different selective mechanisms. If Ka/Ks < 1, it indicates that the protein-coding genes are undergoing purifying selection. When Ka/Ks > 1, it means that these genes are under probable positive selection, and Ka/Ks =1 indicates neutral evolution. During calculation, the value of Ka/Ks was represented by NA if Ks = 0, and was not considered in our analysis.

### Phylogenetic analyses

Fifteen *Spiraea* species (10 newly sequenced and 5 available species from GenBank) and two species (*Sibiraea angustata* and *Pentactina rupicola*) as outgroups were used for phylogenetic analysis. The complete cp genome, each individual highly variable region and the combined six mutational hotspot regions were used as the datasets. Multiple sequence alignment of each dataset was performed using MAFFT [37]. After the best-fit model was resolved by MODELTEST v.3.7 [41], Bayesian inference (BI) was performed with MrBayes v3.1.2 [42]. Two independent Markov Chain Monte Carlo (MCMC) chains were run, each with three heated and one cold chain for 500,000 generations. Each chain started with a random tree, default priors, and sampling trees every 100 generations, with the first 25% discarded as burn-in. Stationarity was considered to be reached when the average standard deviation of split frequencies was below 0.01. The ML analyses were performed with RAxML v7.2.6 [43]. The ML tree was inferred with the combined rapid bootstrap (1,000 replicates) and searched for ML tree (the “-f a” option). The GTRGAMMA model was used in all the analyses as suggested (RAxML manual).

## Supplementary Information

**Additional file 1:** **Table S1. **The information of *Spiraea *species used in this study.

**Additional file 2:** **Table S2. **Raw data of Ka/Ks value.

**Additionaly file 3:** **Figure S1.** Frequency of six SSR types in each *Spiraea *chloroplast genome.

**Additionaly file 4:** **Figure S2.** The number of four long repeats in *Spiraea *chloroplast genomes.

**Additionaly file 5:** **Figure S3.** Sequence identity plot for the fifteen *Spiraea *chloroplast genomes with *Spiraea aquilegifolia *as a reference.

**Additionaly file 6**: **Figure S4.** Phylogenetic tree of the fifteen *Spiraea *species inferred by maximum likelihood (ML) and Bayesian inference (BI) methods based on the different mutational hotspot segments. (A) *rpl16*, (B) *rpoB*-*psbM*, (C) t*rnG-UCC*-*atpA*, (D) *ycf1*, (E) *ψycf1*-t*rnL-UAG *and (F) *trnH-GUG*-*psbA*. Numbers at nodes correspond to ML bootstrap percentages and BI posterior probabilities.

## Data Availability

The datasets generated and analyzed during the current study are available in the NCBI GenBank database (https://www.ncbi.nlm.nih.gov/genbank/) and the accession numbers are listed in Table 1. The raw data of ten *Spiraea* cp genomes can be available in the NCBI SRA database and the accession numbers of each species are as follows: SRR22387745 for *S. salicifolia*, SRR22387744 for *S. aquilegiifolia*, SRR22387743 for *S. chinensis*, SRR22387742 for *S. elegans*, SRR22387741 for *S. henryi*, SRR22387740 for *S. hirsuta*, SRR22387739 for *S. tianschanica*, SRR22387738 for *S. mongolica* var. *tomentulosa*, SRR22387737 for *S. purpurea*, and SRR22387736 for *S. japonica*.

## Abbreviations

cp: Chloroplast
π: Nucleotide diversity
SSR: Simple sequence repeat
LSC: Large single copy
SSC: Small single copy
tRNA: Transfer RNA
rRNA: Ribosomal RNA
PCG: Protein-coding gene
CDS: Coding sequence
Ka: Nonsynonymous mutation rate
Ks: Synonymous mutation rate
IR: Inverted repeat
ITS: Internal transcribed spacers
NGS: Next-generation sequencing
BI: Bayesian inference
ML: Maximum likelihood
MCMC: Markov Chain Monte Carlo
