# Supplementary material for: Characterization and phylogenetic analyses of ten complete plastomes of Spiraea species
Source: BMC Genomics. 2023 Mar 21;24:137. doi: 10.1186/s12864-023-09242-3 (PMC10029230; doi:10.1186/s12864-023-09242-3)
Supplement: Supplementary file 1 — Additional file 1: Table S1. The information of Spiraea species used in this study. [file 12864_2023_9242_MOESM1_ESM.pdf]

Table S1 The information of *Spiraea* species used in this study

Species	Voucher Nubmer	Geographic Origin	Longitude and Latitude	Elevation (m)
Spiraea aquilegiifolia	HuangXW1006	Nei Mongol Autonomous Region	N49°12′<br>E119°42′	624
S. chinensis	10CS1904	Hubei province	N31°43′<br>E110°27′	2137
S. henryi	GanQL1132	Hubei province	N31°50′<br>E109°45′	1035
S. elegans	NiuYL197	Hebei province	N38°18′<br>E114°22′	650
S. hirsuta	Lilan876	Shandong province	N36°17′<br>E118°3′	1012
S. japonica	15CS11508	Sichuan province	N31°54′<br>E107°42′	874
S. purpurea	ZhouZK11324	Sichuan province	N27°9′<br>E101°16′	2686
S. salicifolia	HuangXW1801	Nei Mongol Autonomous Region	N50°48′<br>E119°59′	712
S. tianschanica	16CS12227	Xinjiang Uygur Autonomous Region	N47°55′<br>E88°08′	1035
S. mongolica var. *tomentulosa*	ZuoZh229	Ningxia Hui Autonomous Region	N 38º31′<br>E 106º05′	1142
