# Supplementary material for: Characterization and phylogenetic analyses of ten complete plastomes of Spiraea species
Source: BMC Genomics. 2023 Mar 21;24:137. doi: 10.1186/s12864-023-09242-3 (PMC10029230; doi:10.1186/s12864-023-09242-3)
Supplement: Supplementary file 2 — Additional file 2: Table S2. Raw data of Ka/Ks value. [file 12864_2023_9242_MOESM2_ESM.pdf]

Table S2 Raw data of Ka/Ks value

Category	Gene name	Ka/Ks	Gene name	Ka/Ks
**Photosynthesis pathways**	*atp*A	0.117	*pet*N	0.000
	*atp*B	0.015	*psa*A	0.000
	*atp*E	0.281	*psa*B	0.000
	*atp*F	0.070	*psa*C	0.000
	*atp*H	NA	*psa*I	0.000
	*atp*I	0.132	*psa*J	0.000
	*ndh*A	0.146	*psb*A	0.000
	*ndh*B	NA	*psb*B	0.042
	*ndh*C	0.194	*psb*C	0.000
	*ndh*D	0.067	*psb*D	0.000
	*ndh*E	0.019	*psb*E	0.000
	*ndh*F	0.361	*psb*F	NA
	*ndh*G	0.973	*psb*H	0.000
	*ndh*H	0.169	*psb*I	NA
	*ndh*I	0.057	*psb*J	NA
	*ndh*J	NA	*psb*K	0.000
	*ndh*K	0.197	*psb*L	NA
	*pet*A	0.082	*psb*M	0.000
	*pet*B	0.000	*psb*N	NA
	*pet*D	0.928	*psb*T	NA
	*pet*G	NA	*psb*Z	0.000
	*pet*L	0.000	*rbc*L	0.829
**Transcription and translation**	*rpo*A	NA	*rpo*C1	0.337
	*rpo*B	0.169	*rpo*C2	0.370
**Ribosomal proteins**	*rpl*2	0.190	*rps*4	NA
	*rpl*14	0.037	*rps*7	0.000
	*rpl*16	0.000	*rps*8	0.367
	*rpl*20	0.000	*rps*11	0.000
	*rpl*22	0.363	*rps*12	NA
	*rpl*23	NA	*rps*14	0.086
	*rpl*32	0.000	*rps*15	0.191
	*rpl*33	0.073	*rps*16	0.225
	*rpl*36	0.000	*rps*18	0.000
	*rps*2	0.019	*rps*19	0.036
	*rps*3	0.000		
**Other genes**	*acc*D	0.381	*infA*	0.136
	*ccs*A	0.201	*ycf*1	0.356
	*cem*A	0.216	*ycf*2	0.375
	*clp*P	0.000	*ycf*3	0.000
	*mat*K	0.441	*ycf4*	0.000
