# Supplementary figures and images for: Characterization and phylogenetic analyses of ten complete plastomes of Spiraea species
Source: BMC Genomics. 2023 Mar 21;24:137. doi: 10.1186/s12864-023-09242-3 (PMC10029230; doi:10.1186/s12864-023-09242-3)

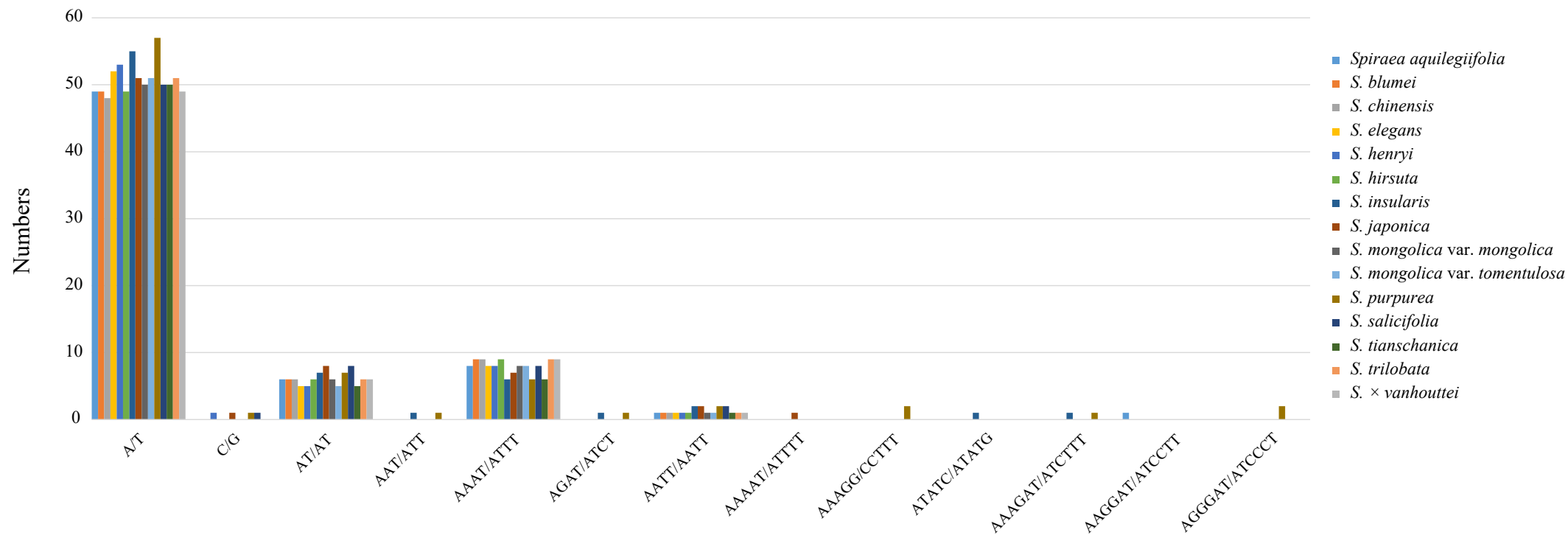

Figure S1 Frequency of six SSR types in each *Spiraea* chloroplast genome.

Supplement: Supplementary file 3 — Additionaly file 3: Figure S1. Frequency of six SSR types in each Spiraea chloroplast genome. [file 12864_2023_9242_MOESM3_ESM.pdf]

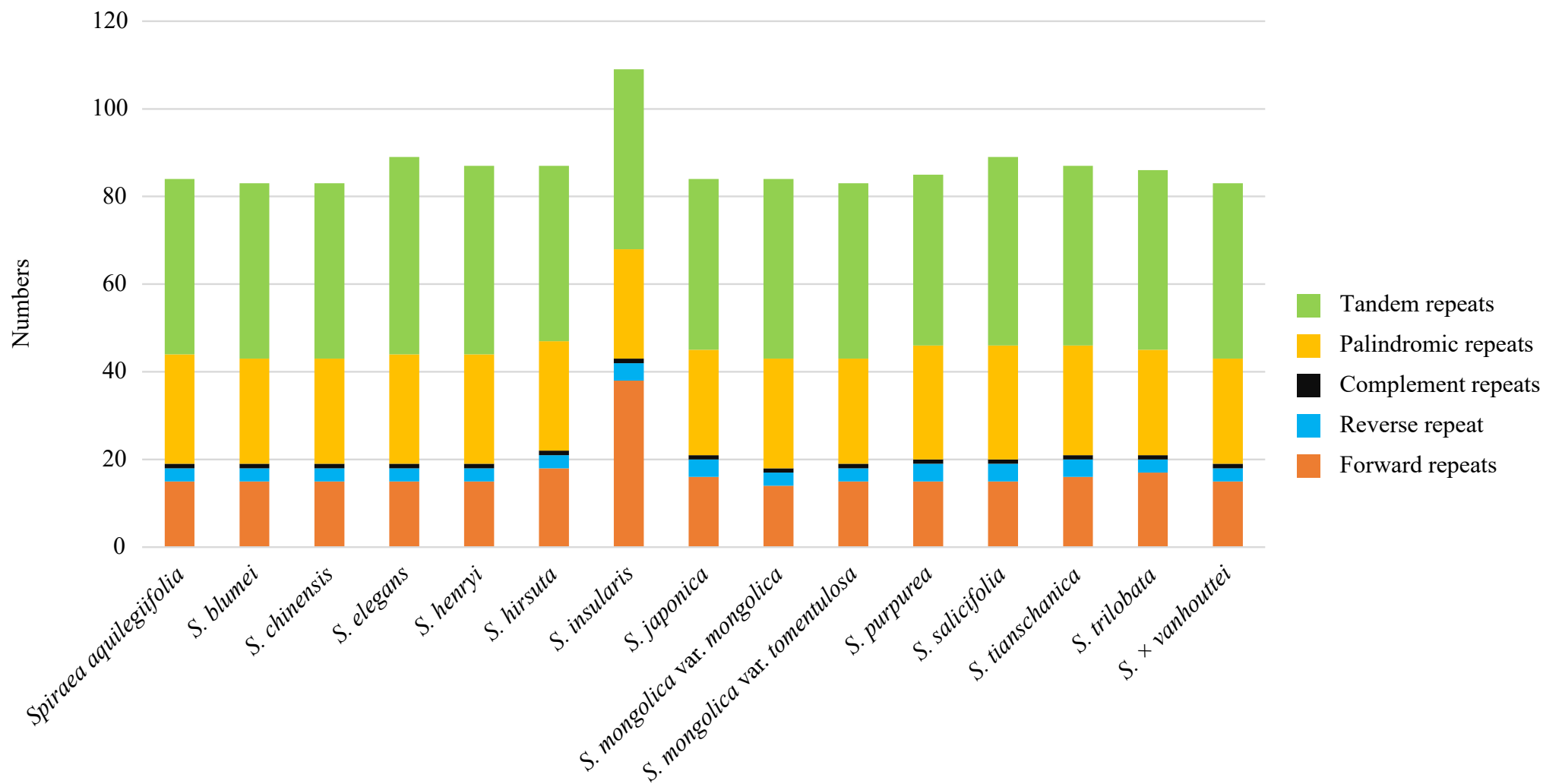

Figure S2 The number of four long repeats in *Spiraea* chloroplast genomes.

Supplement: Supplementary file 4 — Additionaly file 4: Figure S2. The number of four long repeats in Spiraea chloroplast genomes. [file 12864_2023_9242_MOESM4_ESM.pdf]
