# Supplementary material for: Characterization and phylogenetic analyses of ten complete plastomes of Spiraea species
Source: BMC Genomics. 2023 Mar 21;24:137. doi: 10.1186/s12864-023-09242-3 (PMC10029230; doi:10.1186/s12864-023-09242-3)
Supplement: Supplementary file 5 — Additionaly file 5: Figure S3. Sequence identity plot for the fifteen Spiraea chloroplast genomes with Spiraea aquilegifolia as a reference. [file 12864_2023_9242_MOESM5_ESM.pdf]

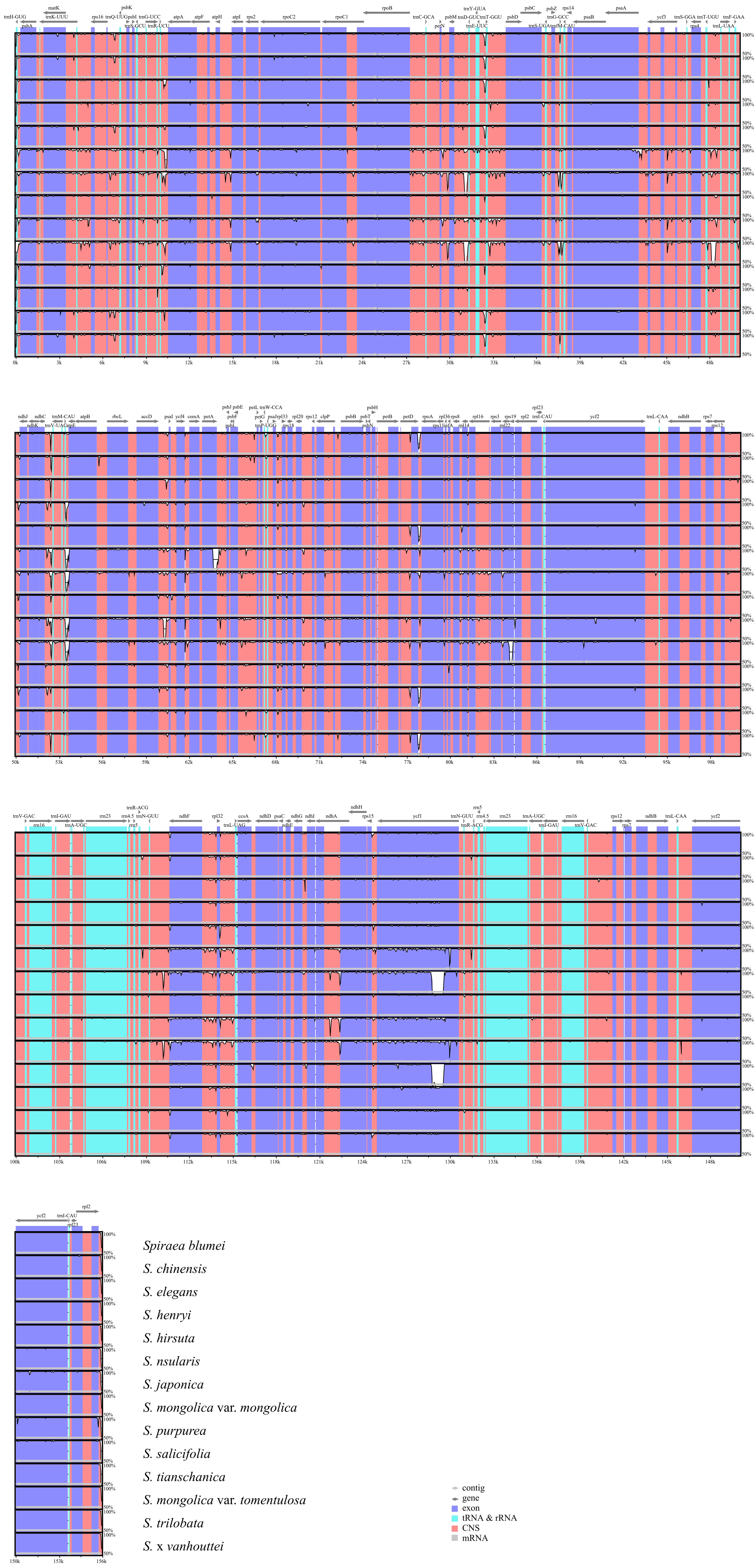

Figure S3 Sequence identity plot for the fifteen *Spiraea* chloroplast genomes with *Spiraea aquilegifolia* as a reference.
