# Supplementary material for: Characterization and phylogenetic analyses of ten complete plastomes of Spiraea species
Source: BMC Genomics. 2023 Mar 21;24:137. doi: 10.1186/s12864-023-09242-3 (PMC10029230; doi:10.1186/s12864-023-09242-3)
Supplement: Supplementary file 6 — Additionaly file 6: Figure S4. Phylogenetic tree of the fifteen Spiraea species inferred by maximum likelihood (ML) and Bayesian inference (BI) methods based on the different mutational hotspot segments. (A) rpl16, (B) rpoB-psbM, (C) trnG-UCC-atpA, (D) ycf1, (E) ψycf1-trnL-UAG and (F) trnH-GUG-psbA. Numbers at nodes correspond to ML bootstrap percentages and BI posterior probabilities. [file 12864_2023_9242_MOESM6_ESM.pdf]

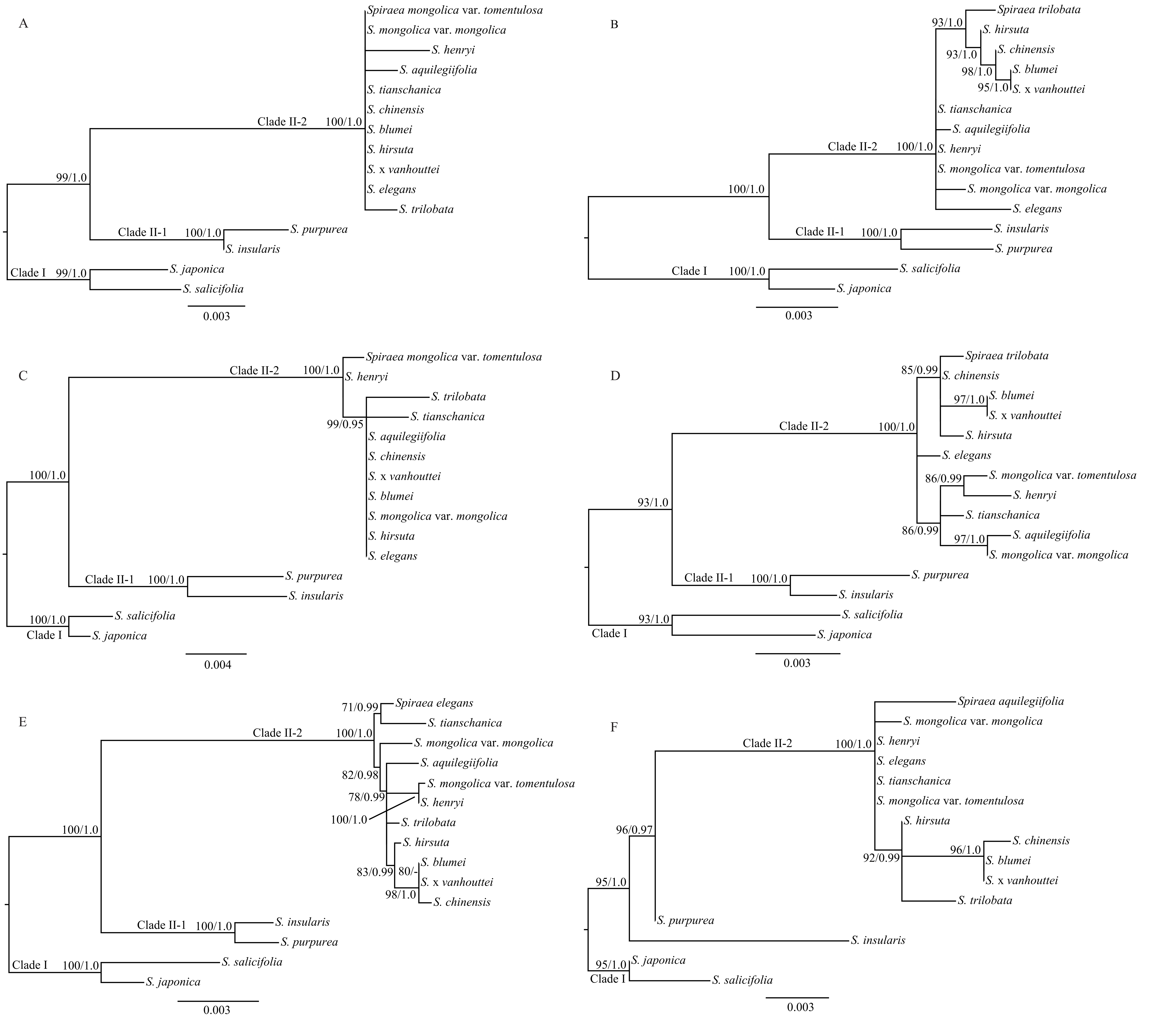

Figure S4 Phylogenetic tree of the fifteen *Spiraea* species inferred by maximum likelihood (ML) and Bayesian inference (BI) methods based on the different mutational hotspot segments. (A) *rpl16*, (B) *rpoB-psbM*, (C) t*rnG-UCC-atpA*, (D) *ycf1*, (E) *ψycf1*-t*rnL-UAG* and (F) *trnH-GUG-psbA*. Numbers at nodes correspond to ML bootstrap percentages and BI posterior probabilities.
